# Constitutive Model and Cutting Simulation of Titanium Alloy Ti6Al4V after Heat Treatment

**DOI:** 10.3390/ma12244145

**Published:** 2019-12-11

**Authors:** Xiaohua Qian, Xiongying Duan

**Affiliations:** 1 Faculty of Electronic Information and Electrical Engineering, Dalian University of Technology, Dalian 116024, China; dxy@dlut.edu.cn; 2 College of Mechanical and Energy Engineering, Ningbo Institute of Technology, Zhejiang University, Ningbo 315100, China

**Keywords:** titanium alloy, split Hopkinson press bar, constitutive equation, Johnson–Cook, stress–stain curves, cutting simulation

## Abstract

As a typical high specific strength and corrosion-resistant alloy, titanium alloy Ti6Al4V is widely used in the aviation, ocean, biomedical, sport, and other fields. The heat treatment method is often used to improve the material mechanical properties. To investigate the dynamic mechanical properties of titanium alloy Ti6Al4V after heat treatment, dynamic compressive experiments under high temperature and high strain rate were carried out using split Hopkinson press bar (SHPB) equipment. The stress–strain curves of Ti6Al4V alloy under different temperatures and strain rates were obtained through SHPB compressive tests. The Johnson–Cook (J–C) constitutive equation was used for expressing the stress–strain relationship of titanium alloy under large deformation. In addition, the material constants of the J–C model were fitted based on the experimental data. An orthogonal cutting simulation was performed to investigate the cutting of Ti6Al4V alloy under two different numerical calculation methods based on the established J–C model using the finite element method (FEM). The simulation results confirm that the adiabatic mode is more suitable to analyze the cutting of Ti6Al4V alloy.

## 1. Introduction

Due to their unique properties such as high specific strength, corrosion resistance, and excellent biocompatibility, titanium alloy materials have increasingly been used in the aviation, marine, biomedical, sport, and other fields [1,2]. Mechanical properties are very important for the application of titanium alloy. Much successful research has been carried out by many scholars to investigate the mechanical properties of titanium alloys [3,4,5,6,7,8]. Filip et al. [3] investigated the effect of lamellar microstructure morphology and phase composition on tensile properties and fracture toughness of titanium alloys after heat treatment. Ivasishin et al. [4] compared the mechanical performances of four titanium alloys in the solution-treated-and-aged condition following thermal mechanical processing. Guo et al. [5] studied the change of microstructure and mechanical properties of titanium alloy TC4-DT after different heat treatment methods. Zherebtsov et al. [6] investigated the microstructure evolution and mechanical properties of two types of titanium alloys under the uniaxial compress process. Dehghanmanshadi et al. [7] analyzed the influence of microstructure morphologies on the mechanical behaviors of titanium alloy using hot compress tests.

However, the processing of metal materials is usually accompanied by high strain and high temperature [9]. The mechanical properties of metals under high temperature and high strain rate are usually different from those under normal temperature and low strain rate. Therefore, analyzing the mechanical behavior of metals under high strain and high temperature is helpful to the study of deformation behavior during machining. The split Hopkinson pressure bar (SHPB) technique is the one most frequently applied in the field of mechanical science to observe the mechanical properties of metals under high strain rate [10,11,12,13,14,15,16,17,18,19,20,21]. Lee and Lin [10] used an SHPB system to systematically study the microstructure change and impact properties of stainless steel 304L. Wu and To [11] studied the chip formation of titanium alloy TC21 in the cutting process based on a series of high-temperature and strain-rate SHPB experiments. Chen et al. [12] adopted SHPB equipment to observe the dynamic mechanical properties of ductile alloys. Kajberg and Sundin [13] presented a split Hopkinson experimental device with an inductive heating source to characterize the mechanical response of steels in manufacturing processes. Hall and Guden [14] investigated the influence of different lubricant conditions on the flow stress–strain of 6061-T651 Al alloy by the split Hopkinson press par. Mylonas and Labeas [15] studied the dynamic mechanical behaviors of aluminum alloy with high strain rate and temperature using SHPB equipment. Taşdemirci et al. [16] analyzed the experimental compressive stress–strain behavior of 316 L stainless steel with a high strain rate using an SHPB experimental system.

Finite element methods (FEMs) have played a very important role in research on the machining process of metals and their application is increasing. The material constitutive model is one of the input conditions of a finite element model, which is very important for the accuracy of numerical simulation results [22]. Johnson and Cook [23] presented a constitutive model which describes the flow stress of materials considering the effects of strain, strain rate, and temperature, and it is very suitable to express the deformation behavior of metals under high strain rate and temperature. This model is widely used as a constitutive equation in machining simulation by many researchers [24,25,26,27,28,29,30], and is often established by the SHPB tests [31,32,33,34,35,36,37]. Wu and Zhang [24] used the Johnson–Cook (J–C) equation as the constitutive model of Ti6Al4V alloy to investigate the milling process using the finite element method. Umbrello et al. [25] studied the effects of J–C constitutive parameters of AISI 316L in an orthogonal cutting simulation on cutting force, chip formation, temperature distribution, and residual stress. Shrot and Baker [26] created an ideal FEM of high-speed cutting using the special J–C parameters for describing the mechanical behavior. Ducobu et al. [27] analyzed the importance of the J–C model parameters and the influence on an orthogonal cutting process of Ti6Al4V alloy.

In this study, we used the split Hopkinson pressure bar (SHPB) to analyze the dynamic behaviors of titanium alloy Ti6Al4V after heat treatment. The temperatures and strain rates for the SHPB experiment on Ti6Al4V alloy cover a wide range. The stress–strain curves were obtained through the high-temperature and strain-rate tests. The J–C constitutive equation was established based on the data from the SHPB tests. The orthogonal cutting simulations of Ti6Al4V alloy were carried out with two numerical models, namely temperature-displacement model and adiabatic model provided by the software Abaqus 6.13 using the established Johnson–Cook material model.

## 2. Materials and Methods

In this study, titanium alloy Ti6Al4V (General Research Institute for Nonferrous Metals, Beijing, China) was used as workpiece material. Before heat treatment, the raw blank of Ti6Al4V alloy was forged and the forging temperature was set at 944 °C. After forging, the cooling method used was air cooling. The material was heated to 989 °C and kept at that temperature for 30 min during the heat treatment process, then air cooling was applied. Because titanium alloy Ti6Al4V is difficult to cut and sticks easily to the tool, the cutting performance can be improved using this heat treatment method.

The mechanical parameters of Ti6Al4V alloy after heat treatment are listed in Table 1.

**Figure 1:**
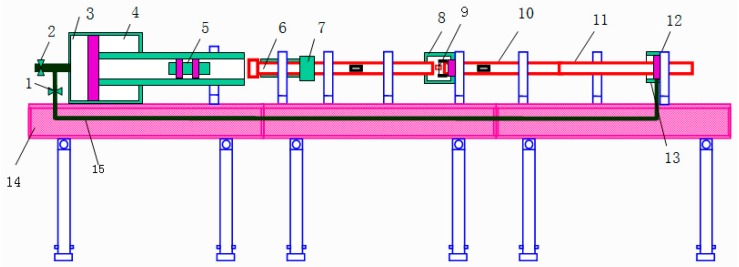
Schematic of high-temperature split Hopkinson pressure bar (SHPB) equipment. 1 Switch; 2 Intake valve; 3 Back air chamber; 4 Front air chamber; 5 Impact rod; 6 Incident bar; 7 Energy absorbing block; 8 Heating furnace; 9 Sample; 10 Transmission rod; 11 Absorbing rod; 12 Piston; 13 Cylinder; 14 Stents; 15 Airway.

To analyze the dynamic mechanical behavior of Ti6Al4V alloy, a series of SHPB experiments under high-temperature and strain-rate conditions were performed. Figure 1 is the schematic of a modified high-temperature SHPB experimental apparatus. In the one-dimensional stress wave theory, the loading process of the SHPB test process can be expressed as follows [38]:

(1) $$\varepsilon_{s}=-\frac{2C_{0}}{l_{s}}\int_{0}^{t}\varepsilon_{R}dt,$$

(2) $$\dot{\varepsilon }=\frac{d\varepsilon }{\mathrm{dt}}=\frac{2C_{0}}{l_{s}}\varepsilon_{R},$$

(3) $$\sigma_{s}=\frac{F_{1}+F_{2}}{{2A}_{s}}=\frac{1}{2}E(\frac{A}{A_{s}})(\varepsilon_{I}+\varepsilon_{R}+\varepsilon_{T})=E(\frac{A}{A_{s}})\varepsilon_{T},$$

where $\varepsilon_{I}$, $\varepsilon_{R}$, and $\varepsilon_{T}$ are the data of incident, reflect, and transmission bar obtained by the strain gages, respectively, and *As* and *L* are the cross-section area and length of the sample. *A* and *E* are the cross-section area and elastic modulus of the pressure bars.

In the Hopkinson bar experiment, the pulse signals in the input and output rods are usually measured by the strain gauge affixed to the rod. When the longitudinal pulse is propagated in the rod, the strain gauge can sense the signal. The signal output by the strain gauge is collected by the measurement and recording system, which requires the transient waveform memory to have a higher sampling rate.

**Figure 2:**
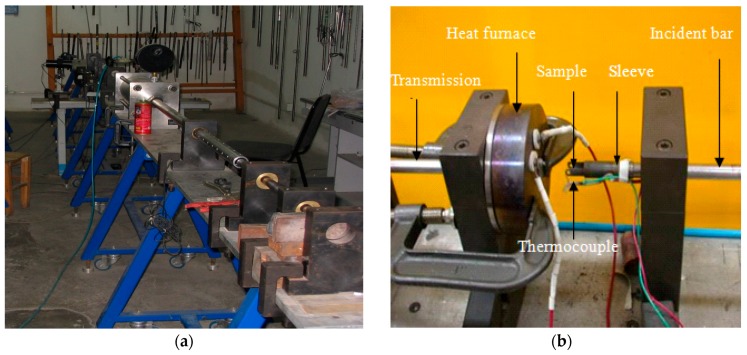
Structure of SHPB apparatus. (**a**) Split Hopkinson press bar test equipment; (**b**) heating furnace and sample fixing.

**Figure 3:**
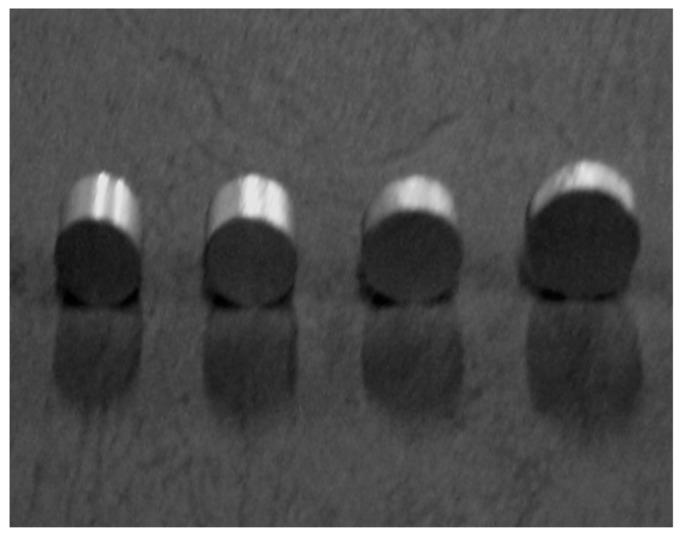
Specimens before and after SHPB tests.

In order to heat the sample during the SHPB experiments of Ti6Al4V alloy, a heating device was added to the SHPB apparatus. In this heating device, one sleeve with two flanges was designed to fix the sample. The thermocouple wire wound around the sleeve was used to measure the sample’s temperature during the testing process. The designed heating device prevents the overheating problem of the incident bar, which improves the test accuracy under high-temperature conditions. The high-temperature SHPB system (The equipment is provided by Northwestern Polytechnical University, Xian, China) with the heating device is shown in Figure 2. The high-temperature SHPB experiments of Ti6Al4V alloy were performed on this modified SHPB system. The samples were fixed between the incident bar and the transmission bar and were heated to the given temperature by the heating device, then pressed under different velocities. The stress–strain curves of Ti6Al4V alloy were obtained through the strain gauges. For the SHPB tests, the Ti6Al4V alloy blank was cut into samples of 5 mm in length and 5 mm in diameter. The samples before and after the compress tests are shown in Figure 3. The parameters of the SHPB experiments are listed in Table 2.

## 3. Stress–Strain Curves Obtained by Hopkinson Press Bar

**Figure 4:**
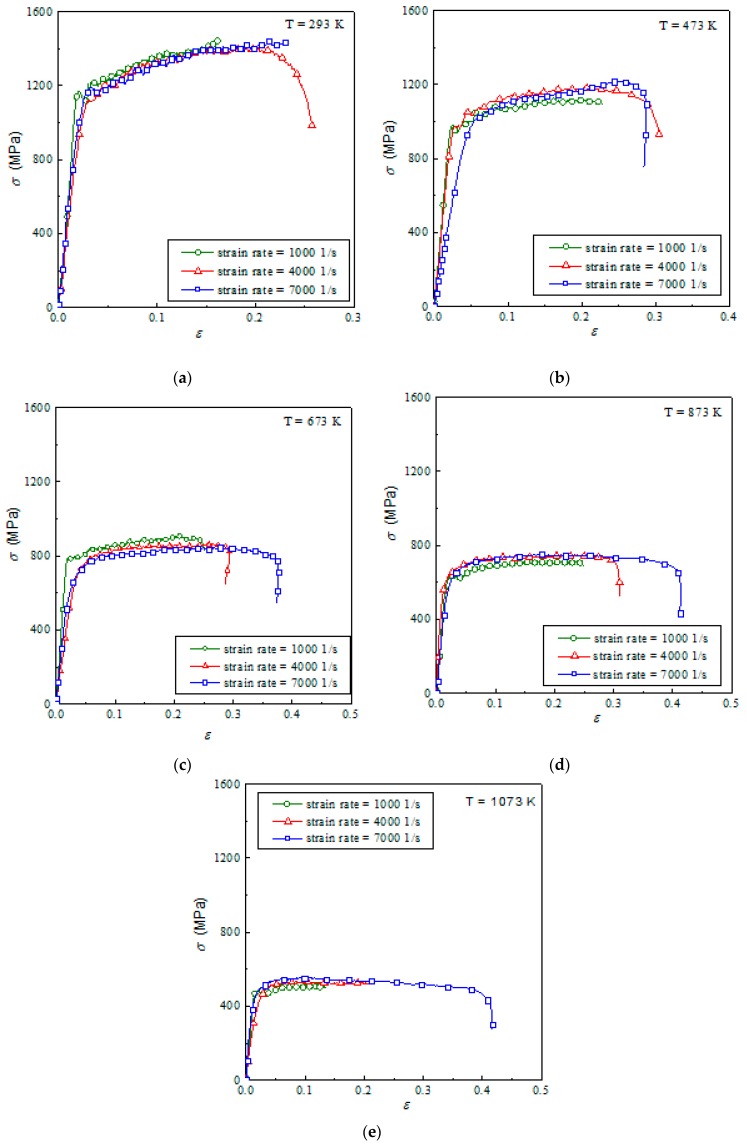
Stress–strain relationship of Ti6Al4V at different temperatures: (**a**) 293 K; (**b**) 473 K; (**c**) 673 K; (**d**) 873 K; (**e**) 1073 K.

Figure 4 presents the effect of strain rate on the true stress–strain curves of Ti6Al4V alloy. This figure shows that the strain rate has a slight influence on the flow stress of Ti6Al4V alloy at the same temperature. With the increase in strain rate, the flow stress and strain hardening rate of the material change little with the strain rate. This proves that the strain hardening effect is not obvious, but that the thermal softening effect is very significant.

**Figure 5:**
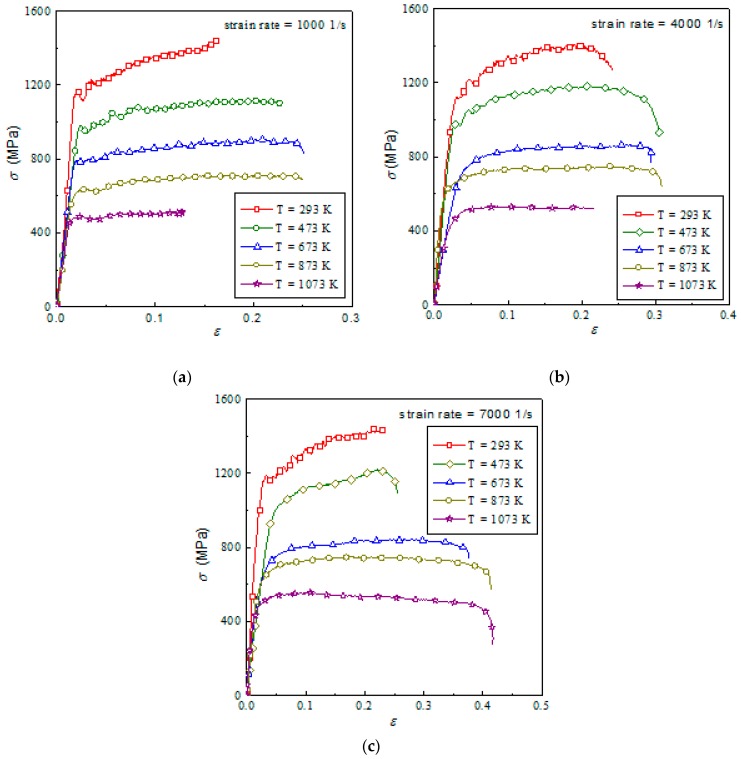
Stress–strain relationship of TI6AL4V at different strain rates: (**a**) 1000 1/s; (**b**) 4000 1/s; (**c**) 7000 1/s.

Figure 5 is the effect of temperature on the true stress–strain curves of Ti6Al4V alloy. Ti6Al4V alloy has a great sensitivity to temperature, and the flow stress continuously decreases with increasing temperature. At lower temperatures, titanium alloy material has obvious strain-hardening characteristics. The figure shows that the flow stress increases gradually with the increasing strain. However, by comparing the experimental results at different temperatures, it was observed that the strain-hardening rate of materials gradually decreased when the experimental temperature increased, and a slight flow softening is seen on the real stress–strain curve at 1073 K. This is because the strain hardening and strain softening caused by the adiabatic effect occur simultaneously after the material yields, which is reflected in the stress–strain curve. At high temperature, the strain softening predominates and the flow stress decreases with the increasing strain.

## 4. Material Model

The classic J–C material law constitutive equation is used as the material model of Ti6Al4V alloy after heat treatment. This model is often used to describe the dynamic behaviors at high strain rate and temperature. The flow stress in this model is expressed as follows:

(4) $$\sigma =(A+B\varepsilon^{n})(1+C\ln\frac{\dot{\varepsilon }}{{\dot{\varepsilon }}_{0}})\left[1-{\left(\frac{T-T_{r}}{T_{m}-T_{r}}\right)}^{m}\right],$$

where ε is equivalent plastic strain, $\dot{\varepsilon }$ is equivalent plastic strain rate, ${\dot{\varepsilon }}_{0}$ is reference strain rate, and *T*, *Tm*, and *Tr* are material, melting, and room temperatures, respectively. *A*, *B*, *C*, *n*, and *m* are the J–C constants.

For the J–C constants of Ti6Al4V alloy, the calculation process is as follows:

(5) $$\sigma =A+B\gamma^{n}.$$

A is equal to the initial yield stress of the material at $\dot{\varepsilon }=1/s$ and $T=T_{r}$, which can be read directly from the true stress–strain curve.

$B\gamma^{n}$ is the description of the strengthening section of the stress–strain curve. Therefore, as long as the collection point of the strengthening section is drawn on the double logarithmic coordinate paper, *B* and *n* can be determined by the following equation:

(6) lnσ=lnB+nlnγ.

The above equation is a line with intercept lnB and slope n on the double-log paper, so n can be expressed as follows:

(7) $$n=\frac{d(\ln\sigma )}{d(\ln\gamma )}=\frac{\Delta \ln\sigma }{\Delta \ln\gamma }.$$

For the equation $\sigma =1+C\ln{\dot{\gamma }}^{*}$, it can be directly plotted on the semi-logarithmic coordinate paper to represent a line with intercept 1 and slope C, and the strain-rate sensitivity coefficient C can be expressed as follows:

(8) $$C=\Delta \sigma /\Delta \ln{\dot{\gamma }}^{*}.$$

For $\sigma =1-T^{*m}$, when $T^{*}>>1$, $\sigma =T^{*m}$, therefore

(9) $$\ln\sigma =m\ln T^{*}.$$

It represents a line on a piece of even coordinate paper, therefore

(10) $$m=\frac{\Delta \ln\sigma }{\Delta \ln T^{*}}.$$

Based on the SHPB test data, the J–C material constitutive model of Ti6Al4V alloy after heat treatment was obtained. Table 3 shows the J–C constants.

**Figure 6:**
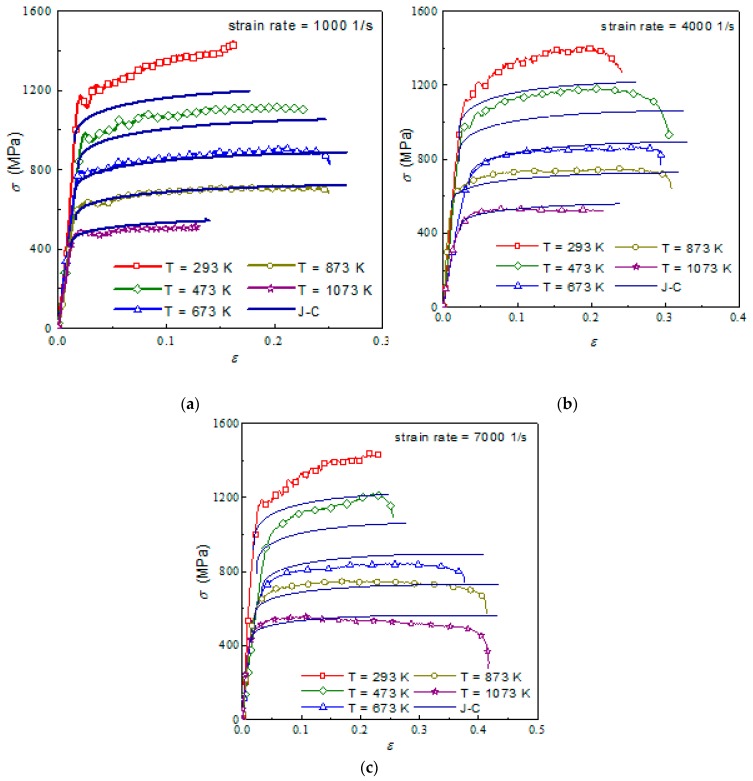
Comparison of JC model and experiment of Ti6Al4V at different strain rates: (**a**) 1000 1/s; (**b**) 4000 1/s; (**c**) 7000 1/s.

Figure 6 presents the comparison of the J–C model and the experimental curves of titanium alloy Ti6Al4V after heat treatment. The J–C model of titanium alloy is very consistent with the experimental results of the high strain rate. At lower temperatures, the J–C model’s simulation results are somewhat different from the experimental ones because of the high strain hardening rate of the materials. However, as the temperature increases, the experimental results become increasingly consistent with the predicted results of the model.

## 5. Cutting Simulation of Ti6Al4v Alloy

**Figure 7:**
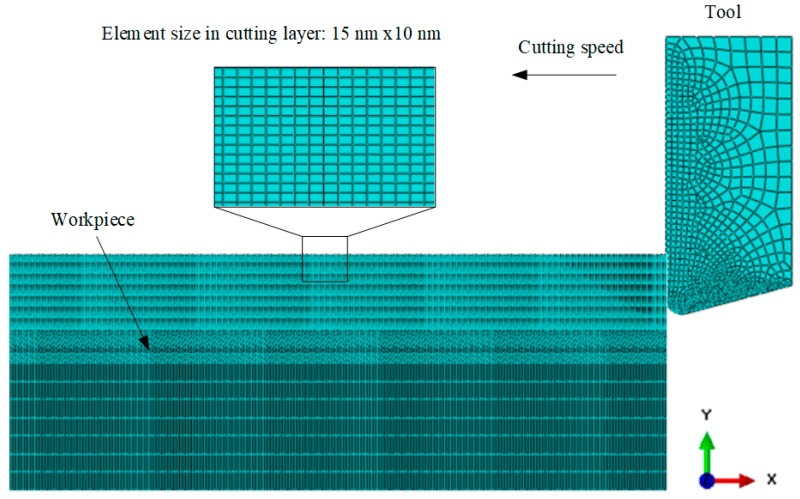
Finite element model of orthogonal cutting process.

An orthogonal cutting model of Ti6Al4V alloy was developed based on the two finite element formulations, namely temperature-displacement (T-D) and adiabatic (AD) modes, provided by the software ABAQUS. The finite element model of the cutting simulation is shown in Figure 7. The workpiece’s dimensions were 5 mm × 1 mm. To enhance the computation efficiency, the meshes in the cut layer of the workpiece were refined. The element type was CPE4RT and the number of workpiece elements was 42,300. The tool was set as a rigid body. The cutting environment was dry cutting. The frictional coefficient obtained by the frictional test was 0.23. The initial temperature was set at 293 K. The cutting simulation’s parameters are listed in Table 4.

**Figure 8:**
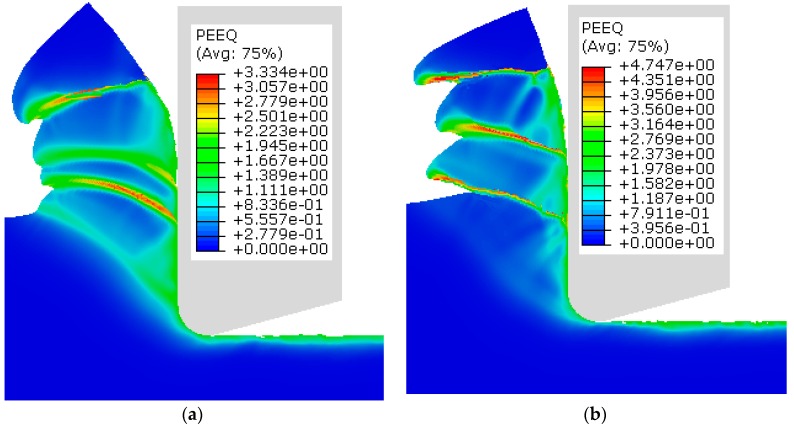
Equivalent plastic strain (PEEQ) for the two numerical models: (**a**) Dynamic temperature-displacement, explicit (T-D); (**b**) Dynamic, explicit, adiabatic (AD).

The cutting simulations of Ti6Al4V alloy were performed using the J–C model established in this paper. The distribution of the equivalent plastic strain (PEEQ) in the cutting simulation at two numerical modes is shown in Figure 8. A higher plastic strain value at the shear band was observed in the adiabatic model when compared to the temperature-displacement model. This is because the work material’s deformation energy in the adiabatic model only focuses on the shear region. There was no heat transfer between the rake face and the chip in the adiabatic simulation. Consequently, the gradually increasing stress led to an increase in plastic strain compared to the temperature-displacement model. In addition, the adiabatic effect in the adiabatic mode caused the more obvious chip serration.

**Figure 9:**
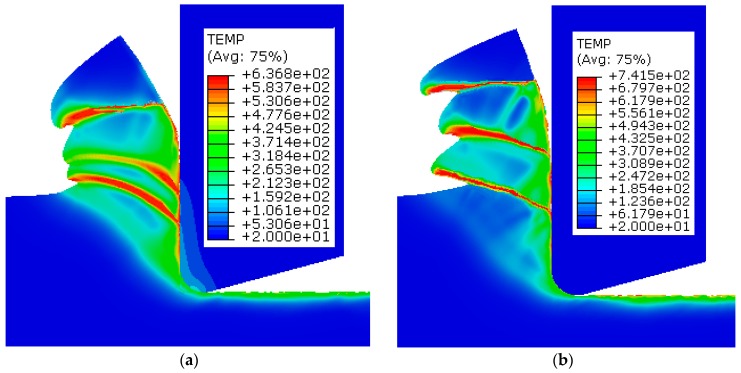
Temperature comparison of the two numerical modes: (**a**) Dynamic temperature-displacement, explicit (T-D); (**b**) Dynamic, explicit, adiabatic (AD).

The temperature distribution in the cutting simulation was compared between the two numerical modes and is shown in Figure 9. The adiabatic mode predicted higher temperatures at the shear band and machined surface compared to the temperature-displacement mode. This can be also attributed to the fact that there was no heat conductivity in the adiabatic simulation. Due to the completely adiabatic calculation, a higher temperature of 741 °C was observed at the shear band when compared to 636 °C of the temperature-displacement mode. This occurs because the cutting heat cannot be transferred to the cutting tool, and no temperature plot occurred in the cutting tool.

Table 5 presents a comparison of the cutting simulation data under the two numerical modes. As can be seen from the table, the thermal softening in the shear region caused by the adiabatic effect made the material slip easily. This occurs because, in the adiabatic simulation, the heat in the shear zone did not have time to transfer out, so the temperature of the shear zone in the AD simulation is higher than that in the T-D simulation. Shear slippage occurs earlier in the AD simulation, the chip pitch and the primary cutting force being smaller than in the T-D simulation.

## 6. Conclusions

Ti6Al4V alloy is the most widely used titanium alloy in the aviation, ocean, biomedical, and sport fields. To improve the material mechanical properties, titanium alloy is often heat treated. Many SHPB compressive experiments at high temperature and strain rate were carried out to investigate the dynamic mechanical properties of titanium Ti6Al4V alloy after heat treatment. The stress–strain relationship of Ti6Al4V alloy under different temperatures and strain rates was obtained through the SHPB tests. The J–C constitutive model of Ti6Al4V alloy was established. An orthogonal cutting FEM was used to investigate the cutting of Ti6Al4V alloy under two different numerical calculation methods based on the established J–C model. The results between the two modes were analyzed through simulation. The results prove that the adiabatic analysis is more suitable to investigate the cutting process of Ti6Al4V alloy because the heat conductivity of this alloy is very low.

## Figures and Tables

**Table 1:** Mechanical parameters of Ti6Al4V.

Properties	Value
Hardness (HRC)	30.6
Density (kg/m^3^)	4450
Elastic Modulus (GPa)	112.5
Yield Strength (MPa)	773.9
Heat Conductivity (W/mk)	3.85
Linear Thermal Expansion (10^−6^/°C)	6.5

**Table 2:** SHPB experimental conditions of Ti6Al4V alloy.

Parameters	Values
Temperature	293 K, 473 K, 673 K, 873 K, and 1073 K
Strain rate	1000 1/s, 4000 1/s, and 7000 1/s

**Table 3:** J–C parameters of Ti6Al4v alloy.

A (MPa)	B (MPa)	n	C	M
874	583	0.316	0.003	0.95

**Table 4:** Cutting conditions of Ti6Al4V alloy.

Parameters	Values
Depth of cut (mm)	0.2
Cutting speed (m/min)	60
Insert material	Carbide
Rake angle (^o^)	0
Clearance angle (^o^)	15
Radius of tool tip	0.05 mm
Cutting environment	Dry cutting

**Table 5:** Comparison of the simulation with the two numerical modes.

	Chip Pitch (µm)	Primary Cutting Force (N)
T-D	35	187
AD	26	175
